# Comparison of D2 and D2 plus radical surgery for advanced distal gastric cancer: a randomized controlled study

**DOI:** 10.1186/s12957-019-1572-1

**Published:** 2019-02-06

**Authors:** Pengfei Yu, Yian Du, Zhiyuan Xu, Ling Huang, Xiangdong Cheng

**Affiliations:** Department of Abdominal Surgery, Zhejiang Cancer Hospital, Hangzhou, 310022 China

**Keywords:** Gastric neoplasm, Lymph node dissection, Complications, Metastasis, Prognosis

## Abstract

**Background:**

The optimal extent of lymph node (LN) dissection for advanced distal gastric cancer remains controversial. The present study compared the safety and efficacy of extended LN dissection (D2 plus) with standard D2 radical surgery for advanced distal gastric cancer.

**Methods:**

Eligible patients were enrolled and randomly assigned into two groups: D2 group and D2 plus group. Patients in the D2 group received standard D2 LN dissection, while patients in the D2 plus group received an additional nos. 8p, 12b, 13, and 14v LNs dissection. The clinicopathological and surgical data of these two groups were compared, and the factors that may influence survival were analyzed.

**Results:**

Seventy patients were enrolled, out of which 64 patients were analyzed. There were no significant differences in the operative time, blood loss, and complications between the two groups. In the D2 plus group, the positive rate of the nos. 12b, 13, and 14v LN was 3.1%, 9.4%, and 12.5%, respectively. The positive rate of the no. 13 LN correlated with the duodenal involvement, while the positive rate of the no. 14v LN correlated with no. 6 LN metastasis. The survival analysis indicated that among patients with duodenum involvement, the 3-year disease-free survival rate of the D2 plus group was significantly better than that of the D2 group.

**Conclusion:**

Duodenum involvement and positive no. 6 LN were high-risk factors of advanced distal gastric cancer. D2 plus radical surgery turned out to be safe and feasible, and may improve the prognosis of these patients. However, further clinical trials are still warranted.

**Trial registration:**

This study is registered with ClinicalTrials.gov as NCT01836991, registered on 22 April 2013.

**Electronic supplementary material:**

The online version of this article (10.1186/s12957-019-1572-1) contains supplementary material, which is available to authorized users.

## Background

Gastric cancer is the fifth most common cancer and the third most common cause of cancer death worldwide [1]. Although chemotherapy and targeted therapy have improved the prognosis of advanced gastric cancer, radical surgery remains the most effective treatment strategy [2, 3]. Recurrence or metastasis after radical resection of gastric cancer is frequent with lymph node (LN) metastasis being one of the important reasons [4]. D2 dissection has been regarded as the standard surgical procedure for advanced gastric cancer. However, controversy remains over the extent of LN dissection in gastric cancer with different tumor stages and tumor locations.

Studies have reported that distal gastric cancer can metastasize to the no. 8p, 12b, 13, and 14v LNs due to the specificity of lesions and biological behavior [5–7]. Therefore, determining whether these lymph nodes should be included in the scope of LN dissection (D2 plus) is important. According to the Japanese gastric cancer treatment guidelines, the nos. 13 and 14v LNs are classified as M1 and are not required for D2 radical dissection [8]. However, some patients with metastasis to nos. 13 and 14v LNs had long-term survival after D2 plus LN dissection. Therefore, the clinical significance of D2 plus 8p, 12b, 13, and 14v LN dissection cannot be completely denied in patients with advanced distal gastric cancer. Further studies are warranted to clarify the characteristics of LN metastasis and its impact on prognosis.

The present study aimed to compare the safety and efficacy of D2 and D2 plus radical surgery and determine the subgroup of patients with advanced distal gastric cancer who are likely to benefit from D2 plus LN dissection.

## Methods

### Study design and patients

This is a randomized controlled, prospective, phase II study conducted at Zhejiang Cancer Hospital (Hangzhou, China) in accordance with the Helsinki Declaration and the good clinical practice guidelines. The study protocol was approved by the Institutional Ethics Review Board of Zhejiang Cancer Hospital (approval number: [2013]-01-50). A written informed consent was obtained from each study participant. This trial was registered with ClinicalTrials.gov (#NCT01836991).

Enrollment criteria were as follows: (1) patients with advanced distal gastric adenocarcinoma (including duodenum involvement), which was pathologically confirmed by endoscopic biopsy; (2) absence of distant metastasis; (3) age 18–80 years and Eastern Cooperative Oncology Group (ECOG) performance status of 0–2; (4) good general condition with a life expectancy of > 6 months; (5) patients with alanine transaminase (ALT) and aspartate transaminase (AST) levels less than two times the upper limit of the normal range (ULN), serum total bilirubin < 1.5 times the ULN, serum creatinine < 1.25 times the ULN, platelet counts > 75,000/L, absolute granulocyte counts > 1500/L, hemoglobin levels > 90 g/L, and normal electrocardiogram; (6) fit to receive R0 surgery with D2 or D2 plus lymphadenectomy; (7) did not receive neoadjuvant chemotherapy and other treatments before the surgery.

The exclusion criteria were as follows: presence of a synchronous or metachronous (within 5 years) malignancy; pregnant or lactating patients; severe drug hypersensitivity; mental abnormalities; the systemic administration of corticosteroids; severe respiratory disease; uncontrolled hypertension, diabetes mellitus, and heart disease.

### Surgery

All the eligible patients were enrolled and randomly assigned into two groups: D2 group and D2 plus group. All the surgeons who participated in the present trial had significant experience in gastric cancer surgery, particularly open distal gastrectomy (at least 50 procedures). According to the Japanese gastric cancer treatment guidelines [8], D2 radical surgery (D2 group) for distal gastric cancer refers to the dissection of nos. 1, 3, 4sb, 4d, 5, 6, 7, 8a, 9, 11p, and 12a LNs. D2 plus radical gastrectomy (D2 plus group) refers to the additional en bloc dissection of hepatoduodenal ligament LNs along the common bile duct (no. 12b), posterior LNs along the common hepatic artery (no. 8p), LNs behind the head of the pancreas (no. 13), and LNs along the superior mesenteric vein (no. 14v) (Additional file 1). For patients with duodenum involvement, Billroth II reconstruction was usually performed and the lower margin was ensured to be negative on frozen section. Patients who required combined pancreaticoduodenectomy were not included in the present study.

### Analysis

The primary endpoint was the safety of radical surgery, and the secondary endpoints were 3-year disease-free survival (DFS) and overall survival (OS).

The following parameters were recorded: patient age and gender, tumor location, histological type, surgical procedure, postoperative complications, depth of tumor invasion, and the number of retrieved LNs. The characteristics of the tumor were recorded according to the Japanese gastric cancer classification (3rd edition) [9] and Union for International Cancer Control (UICC) TNM classification (7th edition) [10]. Complications were defined as any deviation from the normal postoperative course and classified according to the Clavien-Dindo severity classification (CDSC) [11]. Surgical data and the characteristics of positive LNs were compared between the D2 and D2 plus group, and the clinicopathological factors that may influence survival were also analyzed.

### Follow-up

All the patients received adjuvant chemotherapy based on platinum and 5-flurouracil (5-FU) for 4–6 cycles. Patients underwent routine follow-ups after surgery once every 3 months within 2 years, and once every 6 months for 2–5 years. The last follow-up was obtained in April 2018. Postoperative survival time was calculated from the time of radical gastrectomy to the end of the last follow-up or death.

### Statistical analysis

Mean ± standard deviation or median and range were used to present the continuous data. Student’s *t* test and chi-square test were used to compare continuous and categorical data, respectively. The Kaplan-Meier method was used to generate the survival curves, which were compared by log-rank test. Statistical analyses were conducted using Statistical Package for Social Sciences (SPSS) version 19.0 software (Chicago, IL, USA). All the statistical tests were two sided, and the differences were considered to be statistically significant when the *P* value was < 0.05.

## Results

### Baseline characteristics

From April 2013 to December 2014, 70 patients were enrolled in this study. Among these patients, four patients did not receive surgery, while two patients received combined pancreaticoduodenectomy. A total of 64 patients (32 patients in each group) were included in the analysis. There were 48 males and 16 females with the median age of 59 years (range 25–76 years). Furthermore, 17 patients had stage II disease and 47 patients had stage III disease. The histological types were adenocarcinoma (58 patients), signet ring cell carcinoma (5 patients), and mucinous adenocarcinoma (1 patient) (Table 1).

**Table 1:** Comparison of clinicopathological factors and surgical data between the D2 and D2 plus group

Clinicopathological factors	D2 radical surgery (32 cases)	D2 plus radical surgery (32 cases)	*P* value
Gender			0.564
Male	25	23	
Female	7	9	
Age (years)			0.522
> 50	27	25	
≤ 50	5	7	
Tumor location			0.554
Lower third	30	31	
Middle third	2	1	
Histological type			0.688
Adenocarcinoma			
Poor differentiation	13	15	
Well-moderate differentiation	16	14	
Signet ring cell carcinoma	2	3	
Mucinous adenocarcinoma	1	0	
TNM stage			0.157
II	11	6	
III	21	26	
Reconstruction			0.069
Billroth I	22	14	
Billroth II	8	17	
Roux-en-Y	2	1	
Operation time (min)	163.3 ± 59.1	179.6 ± 60.7	0.351
Blood loss (ml)	158.7 ± 70.4	181.3 ± 86.0	0.436
Number of LNs	34.8 ± 11.4	39.4 ± 12.0	0.217
Positive LNs	4.5 ± 8.5	6.5 ± 7.9	0.509
Postoperative complications			0.785
Pulmonary infection	1	1	
Wound infection	0	2	
Anastomotic leakage	1	1	
Pancreatic fistula	0	1	
Ascites	1	4	
Hospital stay (days)	9.8 ± 2.5	10.5 ± 1.7	0.768

### Surgical results

All 64 patients received R0 resection. Billroth I (*n* = 36) and Billroth II (*n* = 25) were the main reconstruction methods. The mean operative time and blood loss in the D2 plus group were greater than those in the D2 group, but the difference was not statistically significant (mean operative time 179.6 ± 60.7 min vs. 163.3 ± 59.1 min, *p* = 0.351; mean blood loss 181.3 ± 86.0 ml vs. 158.7 ± 70.4 ml, *p* = 0.436). A total of 12 (18.75%) postoperative complications occurred (Clavien-Dindo grade II), including ascites (*n* = 5), pulmonary infection (*n* = 2), wound infection (*n* = 2), anastomotic leakage (*n* = 2), and pancreatic fistula (*n* = 1). However, there was no statistically significant difference between the two groups (Table 1).

In the D2 plus group, the positive rate of the nos. 8p, 12b, 13, and 14v LNs was 0, 3.1%, 9.4%, and 12.5%, respectively (Table 2). The positive rate of the no. 13 LN correlated with duodenal involvement, while the positive rate of the no. 14v LN correlated with no. 6 LN metastasis. However, the positive rate of the no. 12b LN had not correlated with the depth of invasion, LN metastasis, the duodenal involvement, tumor size, histological type, and other clinicopathological factors (Table 3).

**Table 2:** Positive rate of extended dissected LNs in the D2 plus surgery group

	Positive cases	Positive rate (%)
8p	0	0
12b	1	3.1
13	3	9.4
14v	4	12.5

**Table 3:** The relationship between no. 12b, 13, and 14v LNs involvement and clinicopathological factors

Clinicopathological factors	No.12b		*P* value	No.13		*P* value	No.14v		*P* value
	+	−		+	−		+	−	
T category			0.625			0.382			0.304
T1–2	0	6		0	6		0	6	
T3–4	1	25		3	23		4	22	
No. 6 LNs			0.340			0.087			0.045
Negative	0	15		0	15		0	15	
Positive	1	16		3	14		4	13	
Duodenum involvement			0.370			0.039			0.419
No	1	17		0	18		3	15	
Yes	0	14		3	11		1	13	
Tumor size			0.400			0.335			0.135
≥ 5 cm	0	13		2	11		3	10	
< 5 cm	1	18		1	18		1	18	
Histological type			0.723			0.827			0.884
Adenocarcinoma									
Poorly	1	13		2	12		2	12	
Well-moderately	0	14		1	13		2	12	
SRCC	0	3		0	3		0	3	
MAC	0	1		0	1		0	1	
CA19-9 (U/ml)			0.625			0.382			0.732
≥ 37	0	6		0	6		1	5	
< 37	1	25		3	23		3	23	
CEA (ng/ml)			0.662			0.434			0.581
≥ 5	0	5		0	5		1	4	
< 5	1	26		3	24		3	24	
AFP (ng/ml)			0.744			0.558			0.492
≥ 10	0	3		0	3		0	3	
< 10	1	28		3	26		4	25	

*SRCC* signet ring cell carcinoma; *MAC* mucinous adenocarcinoma; *CA19-9* carbohydrate antigen 19-9; *CEA* carcinoembryonic antigen

### Treatment toxicity

Adjuvant chemotherapy consisting of platinum and 5-fluorouracil (5-FU) was well-tolerated in most patients. Grade 3 or 4 toxicity occurred in 13 of 64 patients (20.3%). Among these, the most common hematological toxic effects were leucopenia/neutropenia (10.9%) and thrombocytopenia (3.1%) while the most frequent non-hematological toxic effects were elevation of serum aspartate aminotransferase levels (4.7%) and rash (1.6%). There was no statistically significant difference in treatment toxicity between the two groups (Table 4).

**Table 4:** Grade 3/4 toxic effects in the two groups

Toxic effects	D2 radical surgery (32 cases)	D2 plus radical surgery (32 cases)	*P* value
Hematological			
Leukopenia/neutropenia	4(12.5%)	3(9.4%)	0.689
Thrombocytopenia	1(3.1%)	1(3.1%)	1.000
Non-hematological			
Transaminase elevation	2(6.3%)	1(3.1%)	0.554
Rash	1(3.1%)	0(0%)	0.314

### Survival analysis

**Fig. 1:**
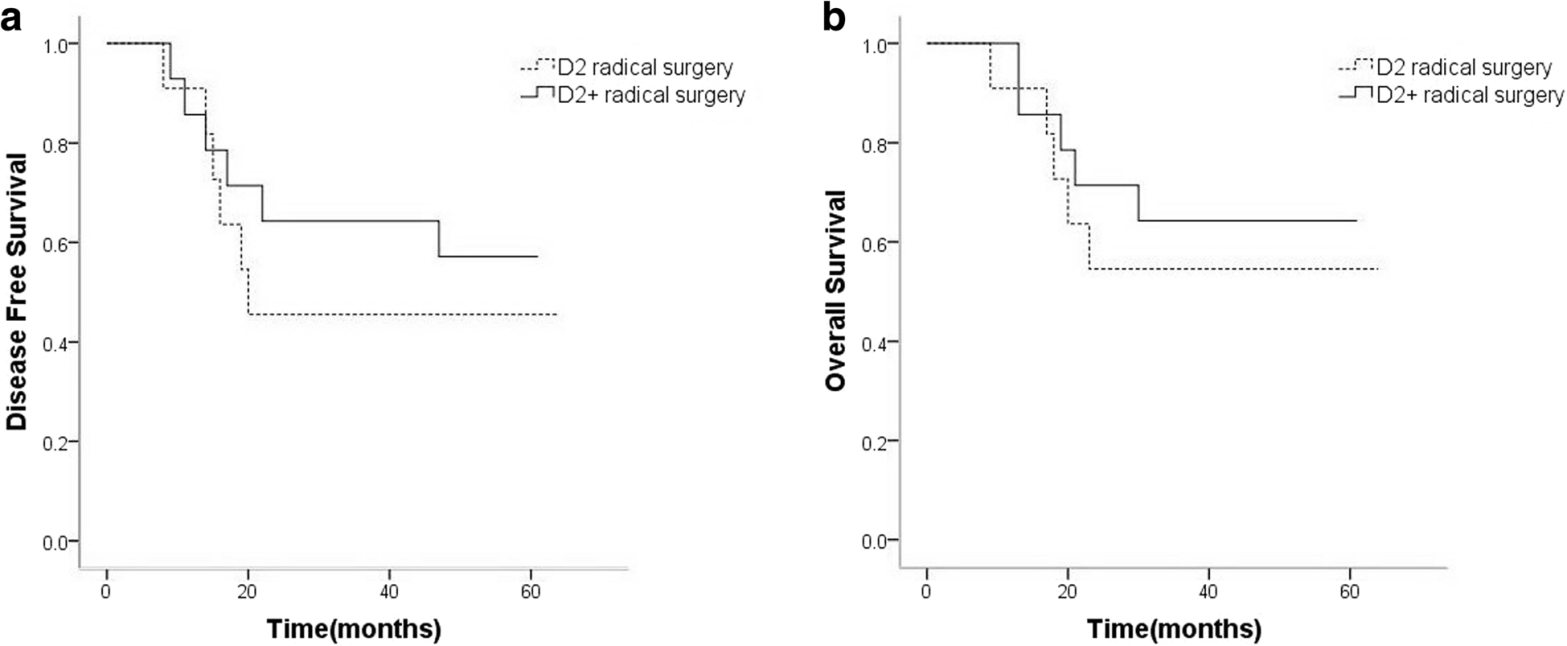
In patients with metastasis to the no.6 LN, the 3-year DFS (**a**) and OS (**b**) of the D2 plus group were higher than that of the D2 group. However, the difference was not statistically significant (*P* > 0.05)

**Fig. 2:**
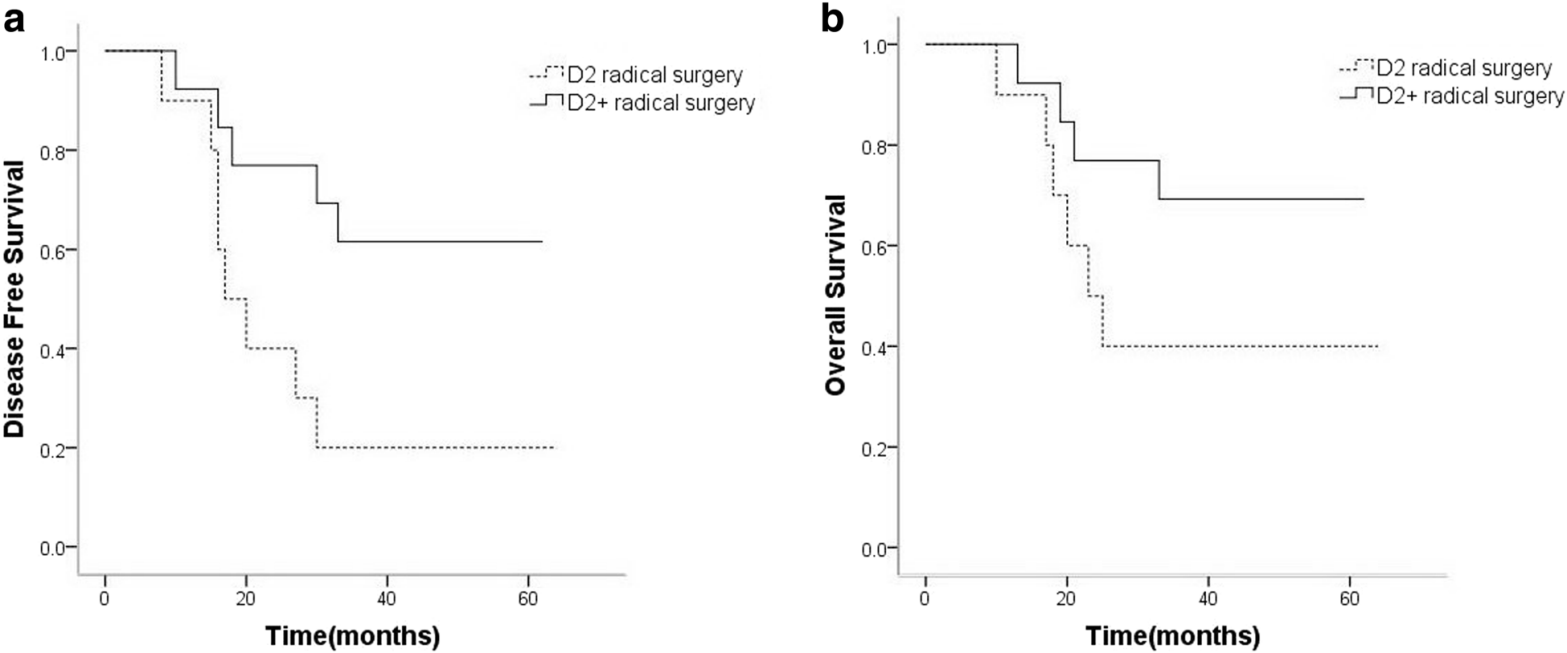
In patients with duodenum involvement, the 3-year DFS of the D2 plus group was significantly higher than that of the D2 group (**a**) (*P* = 0.029). Furthermore, the 3-year OS of the D2 plus group was also higher than that of the D2 group (**b**). However, the difference was not statistically significant (*P* > 0.05)

The mean follow-up period was 39.2 months (10–62 months). A total of 22 patients developed recurrence, while 18 patients died during the follow-up period. The 3-year DFS of the D2 plus group and D2 group were 64.3% and 58.6%, respectively, with no significant difference (*P* = 0.655). The 3-year OS of the D2 plus group and D2 group were similar (71.4% vs. 65.5%, *P* = 0.613). On subgroup analysis of patients with no. 6 LN metastasis, the 3-year DFS and 3-year OS of the D2 plus group were higher than that of the D2 group, but there was no statistical difference (DFS 57.1% vs. 45.5%, *P* = 0.486; OS 64.3% vs. 54.5%, *P* = 0.565) (Fig. 1a, b). For patients with duodenum involvement, the 3-year DFS of the D2 plus group was significantly higher than that of the D2 group (61.5% vs. 20%, *χ²* = 4.763, *P* = 0.029) (Fig. 2a). Furthermore, the 3-year OS of the D2 plus group was also higher than that of the D2 group; however, the difference was not statistically significant (69.2% vs. 40.0%, *P* = 0.133) (Fig. 2b).

## Discussion

Gastric cancer is one of the most common malignancies worldwide [12]. However, its prognosis is very poor despite the advances in the multimodality treatment. Radical surgery has been considered as the main strategy to obtain the exact curative effect for advanced gastric cancer. However, controversy remains on the extent of LN dissection [8]. At present, D2 radical surgery is regarded as the standard surgical procedure for advanced gastric cancer, and there are no consensus on whether an extended LN dissection (D2 plus) should be performed to improve the prognosis [13, 14]. Some researchers have considered that extended LN dissection does not improve the survival and increases the probability of postoperative complications and mortality [15, 16]. However, other studies have shown that extended LN dissection may achieve better results for advanced gastric cancer. Zhang et al. conducted a retrospective study on 567 patients, in which they compared D1 with D3 through D2 lymphadenectomy for gastric cancer [17]. The analysis revealed that in the subgroups of patients with distal gastric cancer, Borrmann II and III, T3 tumors, and clinically LN-positive disease, extended LN dissection could be beneficial without increasing the postoperative complications and mortality. Kumagai et al. reported that in advanced distal gastric cancer with duodenal involvement, there may be survival benefit with the dissection of the no. 12b, 13, 14v, 16a2, and 16b1 LNs [18].

Therefore, a randomized controlled study of D2 radical surgery vs. D2 plus radical surgery was conducted to determine the safety and possible survival benefits of extended LN dissection. This study found that although D2 plus radical surgery was more complicated than D2 radical surgery, there were no significant differences in the mean blood loss and postoperative complications between the two groups. These results are consistent with the previous reports [19, 20]. Therefore, D2 plus radical surgery is safe and feasible.

Studies have shown that advanced distal gastric cancer is prone to metastasize to the nos. 12b, 13, and 14v LNs. Moreover, when distal gastric cancer invades the duodenum, the incidence of metastasis to no. 13 LN can reach up to 23.9% [7]. The probability of metastasis to the no. 14v LN significantly increases in patients with positive no. 6 LN [21]. It was observed that in the cases of D2 plus radical surgery, extended dissected LN had high incidences of metastasis (25%). Also, in patients with duodenal involvement, the local recurrence rate was much higher in the D2 group. Therefore, for advanced distal gastric cancer with high-risk factors such as the involvement of the duodenum or metastasis to the no. 6 LN, merely D2 LN dissection may not be sufficient [19, 22], as positive LNs may remain and cause postoperative recurrence and/or metastasis leading to adverse outcomes.

Our survival analysis revealed that in patients with no. 6 LN metastasis or duodenal involvement, the 3-year OS of the D2 plus group was higher than that of the D2 group, although the difference was not statistically significant, and a separation trend could be observed in the survival curve. The small sample size, single-center study, and short follow-up period were some of the limitations of the present study. Hence, further multi-center large sample studies are required to clarify the survival benefits of D2 plus radical surgery for advanced distal gastric cancer with high-risk factors.

## Conclusion

The present study revealed that for advanced distal gastric cancer with high-risk factors such as the involvement of the duodenum and metastasis to the no. 6 LN, D2 plus radical surgery may be considered. This procedure is safe and feasible, and does not increase postoperative complications, when compared to D2 radical surgery. However, further clinical trials are warranted to validate the findings of this study.

## Additional file

Additional file 1: D2 plus radical surgery for advanced distal gastric cancer. (RMVB 73272 kb)

## Abbreviations

5-FU: 5-flurouracil
ALT: Alanine transaminase
AST: Aspartate transaminase
CDSC: Clavien-Dindo severity classification
DFS: Disease-free survival
ECOG: Eastern Cooperative Oncology Group
LN: Lymph node
OS: Overall survival
UICC: Union for International Cancer Control
ULN: Upper limit of the normal range
